# Assessment of Sports Concussion in Female Athletes: A Role for Neuroinformatics?

**DOI:** 10.1007/s12021-024-09680-8

**Published:** 2024-07-30

**Authors:** Rachel Edelstein, Sterling Gutterman, Benjamin Newman, John Darrell Van Horn

**Affiliations:** Department of Psychology, University of Virginia, 409 McCormick Road Gilmer Hall Room 304, Charlottesville, VA 22904 USA

**Keywords:** Sports concussion, Women’s athletics, Neuroinformatics, Machine learning

## Abstract

Over the past decade, the intricacies of sports-related concussions among female athletes have become readily apparent. Traditional clinical methods for diagnosing concussions suffer limitations when applied to female athletes, often failing to capture subtle changes in brain structure and function. Advanced neuroinformatics techniques and machine learning models have become invaluable assets in this endeavor. While these technologies have been extensively employed in understanding concussion in male athletes, there remains a significant gap in our comprehension of their effectiveness for female athletes. With its remarkable data analysis capacity, machine learning offers a promising avenue to bridge this deficit. By harnessing the power of machine learning, researchers can link observed phenotypic neuroimaging data to sex-specific biological mechanisms, unraveling the mysteries of concussions in female athletes. Furthermore, embedding methods within machine learning enable examining brain architecture and its alterations beyond the conventional anatomical reference frame. In turn, allows researchers to gain deeper insights into the dynamics of concussions, treatment responses, and recovery processes. This paper endeavors to address the crucial issue of sex differences in multimodal neuroimaging experimental design and machine learning approaches within female athlete populations, ultimately ensuring that they receive the tailored care they require when facing the challenges of concussions. Through better data integration, feature identification, knowledge representation, validation, etc., neuroinformaticists, are ideally suited to bring clarity, context, and explainabilty to the study of sports-related head injuries in males and in females, and helping to define recovery.

## Introduction

Historically, sports science research has tended to focus on sports involving primarily male athletes (Asken et al., 2016; Covassin et al., 2003; Koerte et al., 2020; McCrory et al., 2017) where, with a traditionally larger pool of athletes to draw from, male participants have tended to be more accessible for research (D'Lauro et al., 2022; Koerte et al., 2020). Indeed, until 1993 (NIH, 1993) women were not required to be included in National Institutes of Health (NIH) supported clinical research studies. Moreover, societal attitudes have often reinforced gender disparities (Courtenay, 2000), with male athletes receiving more attention and resources in research funding and media coverage (Cowley et al., 2021). Though this has changed moderately in the past three decades, the consequences of this bias are significant, as it limits our understanding of how concussions may manifest differently in female athletes and obscures potentially vital insights into sex-specific risk factors, symptoms, and recovery patterns (Borja et al., 2022). Despite evidence that female athletes experience unique physiological and psychosocial responses to concussions Tierney et al. (2005), medical research has only recently started to recognize this differentiation (Covassin & Elbin, 2011; McAllister & McCrea, 2017; Stone et al., 2017). A recent summary of published research results identified that female athletes need to be adequately represented in sports-related concussions literature (Courtenay, 2000; Covassin et al., 2003). In 2021, a systematic review (D'Lauro et al., 2022) of 161 distinct studies with human participants was conducted to quantify female athlete participation in research. The literature revealed that most studies of sports-related concussions relied on samples that were 80.1% male. Furthermore, 40.4% of these studies did not include female participants. While male and female athletes share some similarities, they display various biomechanical, physiological, and neuroanatomical differences that can adversely affect concussion care and recovery (Chamard et al., 2013). So, while concussion research has seen marked progress, despite these strides, much is still to be learned about the effects of concussions on female athletes.

In what follows, we present a modest, non-exhaustive, scoping review having two particular goals: (i) to examine current and previous literature on neuropathological findings and gaps in female-concussed populations, (ii) to highlight the potential utility of neuroinformatics approaches to effectively characterize, measure, and model the various complexities of sports-related concussion, emphasizing the disparities present in current concussion research as they pertain the female athletes. Furthermore, this appraisal accentuates the need for further advances in neuroinformatics (e.g., multimodal neuroimaging experimental design, data science approaches, etc.) applied concerning female athlete populations, their interpretability, and capability for mitigating sources of assessment and treatment bias. Improved clinical decision making will require both that data collection focuses on female athletes and for analytical techniques to be interpretable in order to link research findings to underlying mechanisms.

### Increasing Recognition of Female Sports Concussions

To address this disparity, national governing bodies such as the National Collegiate Athletic Association (NCAA) and the Department of Defense (DoD) (Broglio et al., 2017) have collaborated with research experts and universities to conduct research that informs injury protocols and has begun to bridge the gap between males and females through the CARE Consortium. One of the main goals of the CARE Consortium was to identify sex-specific factors that aid researchers and healthcare professionals in developing injury prevention and management strategies that are more effective with the hope of developing future strategies that can be tailored to meet each athlete's unique needs

Beyond the NCAA and DoD, other public sector organizations are ardent about promoting equality in concussion research. For example, PINK Concussions (Snedaker, 2015), is a non-profit group dedicated to educating females about brain injuries like sports-related concussions. PINK has been an advocate in creating positive change and innovation by promoting gender-specific, evidence-based approaches for identifying, managing, and supporting women with brain injuries. Additionally, the Concussion Legacy Foundation (Concussion Legacy Foundation, 2007) (CLF), another non-profit organization focusing on promoting female presentation within concussion research, disseminates current research on brain injuries at local, state, national, and international events, as well as through all forms of media.

### Hormonal Implications in Concussion Recovery

There has been a long-standing debate in the field of concussion injury and management regarding the potential biological differences between genders that result in more severe post-injury outcomes (Covassin et al., 2007; Dubol et al., 2021; Gallagher et al., 2018; Kerr et al., 2017; McDevitt & Krynetskiy, 2017; Mollayeva et al., 2018; O'Connor et al., 2017; Wunderle et al., 2014). However, it has been postulated that sex hormones may be a possible explanation (Correia et al., 2010). A few recent reports (Brotfain et al., 2016; Correia et al., 2010) found that women who sustained a mild traumatic brain injury (MTBI) during the luteal phase of their menstrual cycle had a significantly lower quality of life and indicators of health 1 month after discharge than those females who were in the follicular phase of the cycle or were taking hormone contraceptives. Other researchers have suggested that estrogen and progesterone-mediated neuroprotection is thought to be related to their effects on hormone receptors, direct antioxidant effects, effects on astrocytes and microglia, modulation of the inflammatory response to injury, and effects on mediating glutamate excitotoxicity, among others (Fountaine et al., 2019; Green & Simpkins, 2000; Gubbi et al., 2019; Wunderle et al., 2014). However, the true nature of the relationship between increase in estrogen and recovery length post-concussion is poorly understood.

### Chronic Traumatic Encephalopathy Female-Related Research

Diagnosing Chronic Traumatic Encephalopathy (CTE) can be diagnosed within brain tissue post-mortem (Van Horn et al., 2017; Perrine et al., 2017), as this diagnosis requires neuropathologic evidence of perivascular hyperphosphorylated tau (p-tau) aggregates in neurons, with or without astrocytes, typically at the depths of the sulci in the cerebral cortex (Bieniek et al., 2021; McKee et al., 2016; Montenigro et al., 2015). Neuroinformatics may offer a valuable toolkit for shedding light on the phenomenon of CTE in athletes, thought to result from exposed repetitive head impacts (RHI) (Inserra & DeVrieze, 2021; Ling et al., 2017; McKee et al., 2009). It is important to note CTE is a general term for the complex neurodegenerative condition associated with RHI (Van Horn et al., 2017), including multiple concussions (Chamard et al., 2013; McKee et al., 2023). RHI also does not directly always result in a diagnosable concussion, as RHI results come from impact-induced head accelerations that produce tensile and shear strains within the brain's tissues (Van Horn et al., 2017). Moreover, repetitive sub-concussive head impact exposure has been correlated with reducing concussion tolerance (Lakhan & Kirchgessner, 2012). Therefore, a sports-related concussion is defined as a singular injury, whereas RHI is exposure to brain shearing over some time. Neuroinformatics allows for integrating diverse data sources, such as brain imaging, genetic information, and longitudinal clinical data, providing a holistic view of the factors contributing to CTE development. By applying advanced computational methods and ML, researchers would be better able to uncover subtle gender-specific patterns, temporal relationships, and hormonal-specific differences that may be essential in elucidating the pathophysiology of CTE.

Over 97% of CTE cases published have been reported in individuals with known exposure to repetitive head impacts (RHI), with the youngest being a 17-year-old soccer player (Amyot et al., 2015). There has been some discussion surrounding the potential causal relationship between sports-related RHI and CTE. Nevertheless, a new study makes a strong case for any alternative hypotheses that could account for the association between sports-related RHI and CTE (McKee et al., 2023; Mez et al., 2017; Suter et al., 2023). An international brain autopsy study of women who had experienced intimate partner violence further reveals substantial damage in the brain, but no evidence of chronic traumatic encephalopathy (CTE), the neurodegenerative disease recognized among contact sports athletes who sustain repeated head trauma (McKee et al., 2023). These findings become more prevalent when a 17-year-old football athlete exhibited symptoms of the disease after dying at a relatively young age (Amyot et al., 2015; Bieniek et al., 2020; Mez et al., 2017). However, the development of CTE in professional female athletes and the effects RHI has on the plasticity of the female brain needs further investigation.

Despite confirmed diagnoses coming post-mortem, certain diagnostic features are present in over 70% of confirmed CTE cases, which can be classified into three categories: cognitive, behavioral, and mood (Chen, 2018; McKee et al., 2009). One-third of the cases showed symptoms at retirement from the sport, while half showed symptoms within four years of stopping play (Katz et al., 2021). Thus, a clinical criteria was created for the syndrome associated with CTE, known as traumatic encephalopathy syndrome (TES) (Jordan, 2013; Montenigro et al., 2014). TES can be diagnosed based on criteria established by the National Institute of Neurological Disorders and Stroke (NINDS) (Montenigro et al., 2014). The main symptoms of TES include difficulties with thinking and memory and regulating emotions and behavior. People with TES may act impulsively or explosively and have trouble controlling their emotions (Montenigro et al., 2014). The supportive features of CTE include delayed onset of core clinical features, parkinsonism, other motor signs (including amyotrophic lateral sclerosis), depression, anxiety, apathy, and paranoia (Mez et al., 2017). These symptoms have been documented to be present years after RHI exposure has ceased (Chen, 2018; Mez et al., 2017).

The diagnosis of TES does not necessarily confirm the presence of CTE neuropathological changes, such as p-tau accumulation (Jordan, 2013). Rather, TES is a diagnosis of a clinical syndrome that is linked with a history of repetitive brain trauma (Jordan, 2013), clinically evaluated based on provisional diagnostic classifications of ‘probable CTE,’ ‘possible CTE,’ and ‘unlikely CTE’ (Jordan, 1993, 1998; Jack et al., 2011; Jordan, 2013). As research on the clinical presentation of CTE is still in its early stages, establishing significant diagnostic criteria for "probable CTE" based purely on clinical features and course, like the criteria used for probable Alzheimer's Disease dementia in the National Institute on Aging-Alzheimer's Association (NIA-AA) AD diagnostic criteria, is not currently feasible (Covassin et al., 2016).

In leveraging the severity parameter within clinical presentations, controlling for gender could provide valuable insights to the provider and the patient. However, current studies into the pathology of CTE and TES clinical presentation include inadequate female participation or lack of female participation. For instance, one recent study included only one female out of six participants (Jordan, 2013) whereas several others primarily studied TES by examining male amateur and professional boxers (Jordan, 1993, 1998), including no female participants at all. A lack of female representation in sophisticated critical sports medicine studies could lead to misdiagnosis of TES in females, highlighting the significance of adequate female representation in research.

### More Data, More Questions, Greater Need for Data Science?

While neuroimaging techniques constitute useful tools to evaluate in vivo brain structural (MRI and DTI), functional (fMRI, resting state-fMRI) and molecular (PET, SPECT) changes in TBI versus otherwise healthy brain, other data-intensive methods of monitoring brain function, like electroencephalography (EEG) and magnetoencephalography (MEG) are also being applied (Amyot et al., 2015; Mateos-Pérez et al., 2018; Mavroudis et al., 2022). Whole genome-analytics are also being explored to identify gene expression patterns which might predict the differential rates of concussion recovery between males and females (Covassin et al., 2016; Rauchman et al., 2022). Coupled with electronic health records (EHR) research (Tian et al., 2013) and U.S.-based national resources like the Federal Interagency Traumatic Brain Injury Resource (FITBIR), (U.S. Department of Health and Human Services.) there is an increasing need for advanced analytics in order to expedite research and clinical treatment options. Emerging technologies such as ML and AI, in particular, have the potential to revolutionize the understanding of complex brain-health outcomes and AI-based diagnosis tools hold tremendous promise in dissecting the intricate relationship between post-concussion recovery in female athletes, their return to play, as well as their brain health in later life. In particular, implementing advanced ML and AI techniques may enable the evaluation of the comprehensive and integrated perspective of the lucid and consistent picture of the neuroplastic changes associated with hormonal fluctuations during the menstrual cycle. ML may be able to help identify objective biomarkers for concussion as well as useful in predicting concussion recovery. In leveraging these modeling approaches, it may be possible to gain a more nuanced understanding of the complex interplay between hormonal changes and the brain's ability to adapt, thereby paving the way for more personalized and effective treatments. (Ryali et al., 2024).

Rapid increases in clinical and laboratory data collection have demanded a particular emphasis being placed on machine learning (ML) models and artificial intelligence (AI) for improving how neuroimaging data might be modeled and TBI cases classified (Bajwa et al., 2021; Mateos-Pérez et al., 2018; Tamez-Peña et al., 2022). Like many instances of mild traumatic brain injuries (mTBI), concussions do not show visible or specific signs of injury, such as bleeding, and no structural abnormalities are detected in brain imaging with either gender. (Blennow et al., 2012; Davenport & Kalakota, 2019; International Concussion Society 5 years ago, 2019; Mavroudis et al., 2022; McCrory et al., 2008; Raichle, 1998; Rajkomar & Dean, 2019) ML can harness the ability to distinguish these small microstructural differences between two subgroups, as well as relate brain images to clinical or behavioral observations. Moreover, ML-supervised learning, algorithms based on labeled datasets to predict outcomes accurately, can help better understand the recovery process and identify differences in recovery patterns between male and female patients (Bergeron et al., 2019; D’Lauro et al., 2018). Meanwhile, ML unsupervised learning, which uses machine learning algorithms to analyze and cluster unlabeled data sets, can reveal hidden structures in sets of images or uncover sub-populations in large groups of patients (Abraham et al., 2014). By analyzing the microstructural and neurophysiological properties of the brain following a concussion, more personalized and effective treatment interventions could be possible, ultimately improving the quality of care provided to patients. (Abraham et al., 2014; Chamard et al., 2013; Singh et al., 2022; Valera et al., 2021).

In the last decade, using ML techniques is highly beneficial in differentiating gender-specific patterns in neuroimaging data (Chen et al., 2023; Manley et al., 2017). In 2023, researchers from NYU Grossman School of Medicine showed for the first time that ML methods were capable of accurately distinguishing between the brains of male athletes who played contact sports like football versus non-contact sports like track and field. Churchill et al. (2021) also employed ML to detect concussion recovery differences in male and female athletes. Their findings reported no difference in acute symptoms or recovery time, however, all neuroimaging measures showed significant sex differences during recovery (Manley et al., 2017). Similarly, advanced deep learning models were 90% successful at determining whether fMRI scans of brain activity came from a female or a male brain (Chen et al., 2023). These results highlight the utility of ML and AI methods to craft differential neurological biomarkers specific to gender and even sport that do not appear on conventional MRI scans.

Data science-based techniques such as neural networks (NNs) and statistical learning also have major advantages: first, detecting subtle changes, such as sex-related neuropathological differences, may require a particular combination of sensitive neuropsychological and neurological assessments; thus, employing more advanced statistical models is necessary (Castillo, 2023; Dabek & Caban, 2015; Jiarui et al., 2021; Valera et al., 2021). Second is the ability to handle data with small female sample sizes, and female retention defects that are seen in current concussion research. Small sample sizes are common in sports-concussion research and a smaller portion are female. Therefore, ML can help to obtain sufficient results; high-quality within a small female sample size can prove better than a large sample of lower-quality data in the case of statistical ML (Faraway & Augustin, 2018). Third, creating brain imaging pipelines (Deweerdt, 2022) for analyzing brain imaging data in females, which funnels into a repository, can be a helpful source for investigators to collaborate (Castillo, 2023). For example, Leech and colleagues created a well-established algorithm for brain imaging studies in syndromes such as autism (Falcone et al., 2013). The algorithm estimates the performance of other nearby pipelines and datasets from similar subgroups to identify gender-specific clusters (Deweerdt, 2022). Moreover, data science approaches have sophisticated methods of handling missing data through imputation and *post-hoc* analyses to reduce false positive results in female athletes (Kang, 2013; Sterne et al., 2009).

ML has also been utilized to pinpoint biomarkers in other health-related outcomes to construct a predictive model that identified specific patterns within the data. Dabek and Caban (2015) utilized a ML to develop a model that accurately predicted the likelihood of military service members developing posttraumatic stress disorder after a concussion, which was then validated. Notably, deviations in connectivity structures from a ML model predicted brain tumor recurrence up to two months in advance (Nenning & Langs, 2022; Nenning et al., 2020). Similarly, Kampaki (2016) conducted a preliminary classification study, leveraging ML tools to estimate injury recovery time for professional football (soccer) players. Kim (2021) also successfully employed ML algorithms with high accuracy to predict patient outcomes with persistent post-concussive symptoms successfully. Falcone et al. (2013) also employed ML techniques based on vowel sounds extracted from speech recordings and successfully detected concussion incidents with high prediction accuracy.

With the use of ML, it is possible to extract meaningful features from images, which can then be used to generate clinically significant biomarkers specific to gender (Furtner et al., 2017). By leveraging advanced technologies to extract and analyze data from medical images, researchers and healthcare professionals can gain new insights into the underlying mechanisms of various diseases and develop more effective treatment strategies (Fox & Greicius, 2010; Gray et al., 2013). Neuroinformatics within the female brain represents a promising avenue for advancing the field of medicine and improving female patient outcomes (Singh et al., 2022). As such, ML is a powerful tool in detecting and quantifying even the most subtle differences in brain activity between two subpopulations (Nenning et al., 2020).

Deep learning is a more powerful tool for analyzing brain images in ML, which can extract non-linear network structure, realize approximation of complex functions, characterize distributed representation of input data, and demonstrate the powerful ability to learn the essential features of datasets based on a small size of samples (Tian et al., 2018). Differences in) with ML techniques, researchers have revealed subtle changes related to normal brain development (Lasi et al., 2014; Zeng et al., 2016).

### A Role for Neuroinformatics?

In many ways, the field of Neuroinformatics is where brain science meets data science – that is, the intersection of brain-related datatypes, measurements, models, and meta-data suitable for characterization by modern computational data science methods. One area where neuroinformatics has been particularly impactful is in the area of human neuroimaging studies identifying individual biomarkers and combinations of biomarkers, which aim to improve the accuracy of diagnosis of a sports-related concussion or even predict future neurodegeneration associated with repetitive head impacts. It has facilitated cross-modal integration has unveiled the relationships between brain structure and function, shedding light on the neural underpinnings of cognition, behavior, and neurological disorders. Furthermore, a focus on neuroinformatics has played a pivotal role in establishing large-scale, openly accessible databases like the Human Connectome Project (HCP), the Alzheimer's Disease Neuroimaging Initiative (ADNI), and others. These resources have promoted collaboration and knowledge sharing within the scientific community and accelerated the development of advanced analysis methods, such as network neuroscience, which provides insights into the brain's intricate connectivity patterns (Avberšek & Repovš, 2022; Mateos-Pérez et al., 2018; Vieira et al., 2017). Neuroinformatics has been a foremost catalyst for groundbreaking discoveries in human neuroimaging, leading to a deeper understanding of the brain's structure, function, and connectivity and offering new avenues for improving human brain health and addressing neurological challenges.

Neuroinformatics approaches have previously shown promise as valuable analytical tools in addressing the multifaceted challenges that arise from post-concussion neuroimaging research (Abe et al., 2002; Dvorak et al., 2007; Glickstein & Doron, 2008; Lakhan & Kirchgessner, 2012; Lee & Kondziolka, 2005; Prendergast et al., 2015; Shirao et al., 2005; Sullivan et al., 2001; Tian et al., 2018; Zeng et al., 2016). Nevertheless, existing studies are yet to provide consistent results on exploring the difference of brain structure between men and women (Zeng et al., 2016). In a recent study that included female participation, a deep convolutional neural network (CNN) used the convolution kernels to extract the features of the image by using the designed 3D PCNN algorithm. In line with previous work, this study confirmed that gender-related differences exist in the whole-brain fractional anisotropy (FA) images and in each specific brain region. Due to limited research available, more extensive, and diverse datasets are needed to explore these neurological gender differences at a greater length. Continued research, collaboration between data science, clinical neurology, and sports medicine experts, and rigorous testing are essential to ensure that data science-based analytics meet gender-equitable standards and have a promising and reliable future in advancing our understanding and management of sports-related concussions.

One specific challenge concerns how computational methods like ML, deep learning, NNs, etc. are explainable. Model explainability involves describing to humans how and why a ML model’s made a decision. (Castillo, 2023) It should not be unreasonable that a human would like to comprehend an algorithm and its output, and by analyzing the decisions and results of ML models, gain and understanding about the reasoning behind the system’s decision (Samek et al., 2017). This is especially important for ‘black box’ models, which learn directly from data without human guidance. ML models have a slight advantage of having sufficient explainability compared to more deep-learning models (Kang, 2013). But as such models become still more complicated, nuanced, and sophisticated, will this always remain true?

Practitioners of neuroinformatics, having armed themselves over decades with advanced analytic tools specific to a range of brain data types, are essential for making AI methods applied to neuroscience data explainable, particularly needed in contexts such as sports-related concussions. Explainable AI methods aim to make the decision-making process of machine learning models transparent and interpretable to end-users, including researchers, clinicians, and athletes. Neuroinformatics provides a foundation for incorporating domain-specific knowledge into AI models, allowing for the generation of explanations that are not only accurate but also comprehensible to stakeholders with varying levels of expertise. This transparency fosters trust in AI-based diagnostic and prognostic tools for assessing sports-related concussions, facilitating their adoption in clinical practice and athletic settings.

What is more, neuroinformatics promotes rigorous validation and reproducibility of AI algorithms through standardized evaluation protocols and benchmark datasets. By establishing common benchmarks for concussion diagnosis and outcome prediction, researchers can systematically compare different AI approaches and ensure their generalizability across diverse populations and experimental conditions. This systematic validation enhances the reliability and robustness of explainable AI methods, reinforcing their utility in real-world applications.

Furthermore, within the small studies found to have been conducted on concussed female athletes that utilized ML in neuroimaging, these studies exhibited encouraging results in precisely and objectively evaluating gender-specific neuropathological characteristics (Bergeron et al., 2019; Fleck et al., 2021). However, further research is needed to investigate the neural pathological mechanisms involved in female concussions and comprehend the significance of sex differences following a concussion (D’Lauro et al., 2018). Although some promising insights have been discovered, more research using neuroimaging and ML is required to investigate sex differences after sports-related concussions fully (Nenning & Langs, 2022; Singh et al., 2022). Furthermore, advancements in imaging techniques may lead to better comprehension of brain injuries in females in the acute and chronic phases.

Achieving transparency in these models is an imperative for several reasons. First, it enables researchers and healthcare professionals to comprehend and interpret the intricate relationships and patterns of head injury exclusive to one’s biological gender (Bergeron et al., 2019). This understanding is essential for making informed decisions regarding concussion diagnosis, treatment, and prevention strategies that may vary between male and female athletes due to sex-specific differences. Moreover, explainable models enhance the credibility and trustworthiness of findings, making it easier to communicate results to stakeholders, athletes, and their families, ultimately contributing to more effective, tailored, and equitable care for athletes of all genders.

Through integrating diverse datasets, advanced analytical techniques, and ML models, neuroinformatics allows for identifying nuanced sex-specific patterns and distinctions in concussion-related data. It enables researchers to delve into the underlying neural mechanisms, differential symptom presentations, and recovery pathways, which a historical male-centric focus in traditional research may overshadow. Elucidating the underlying mechanisms behind concussions require moving beyond traditional statistical methods not only because of the subtlety of differences, but also because of the limitations inherent in retrospective sport-related concussion populations, where injuries, mechanisms, time to data collection, and physiology of participants can be highly heterogenous and not always available. Moreover, neuroinformatics facilitates the creation of more balanced, inclusive datasets, ensuring equitable representation of brain injuries in male as well as in female athletes. On the other end of the analysis trajectory, neuroinformatics encompasses advanced statistical methodologies which incorporate variability along multiple dimensions, enabling the inclusion of a diverse array of sports, for example, where concussions may result from different impact types (i.e. from another player, playing surface, rotational/torque/shearing, or impacts from athletic equipment). This is an especially important consideration for research in female athletes since a great deal of concussion work has been performed in American football, a sport where concussive forces can be very different than those experienced in sports popular with female athletes. As a result, the approach typically embraced in neuroinformatics has put a premium on the accuracy and interpretability of findings. It has long sought to ensure that the insights gained from analysis of brain data types are universally applicable, make sense clinically, and are sound with respect to foundational neuroanatomy. Adopting such a mindset for dealing with data that affects male subjects differently from female subjects, will ultimately contribute to more equitable and effective concussion management strategy specifically tailored to each athlete, inclusive of the athlete’s sex or gender (International Concussion Society, 2019).

In summary, neuroinformatics provides a solid foundation for enhancing the explainability of AI methods applied to neuroscience data, particularly in the context of sports-related concussions, and those factors which contribute to brain health in female athletes. By facilitating data integration, feature extraction, knowledge representation, model transparency, and validation, neuroinformaticists, with their vast knowledge and experience, are just the community who can help bring clarity, context, and explainability to the modeling needed for vexing problems like differentiating male and female sports-related head injuries, mapping their trajectories, and unambiguously defining what recovery looks like.

## Discussion

As contact sports have traditionally been male-dominated, they have been recognized as being more prone to head injuries. However, research suggests that females may be more vulnerable to sports-related concussions (Covassin & Elbin, 2010, 2011; Covassin et al., 2003; O’Connor et al., 2017), even when participation rates are considered. Although the number of reported cases of CTE in females is currently limited, it is vital to consider the potential risks associated with sports participation for both genders, particularly considering the growing body of evidence on the subject (Bieniek et al., 2020; Mez et al., 2017). CTE is a distinctive neurodegenerative disorder which is said to be triggered by repeated brain injury, frequently sustained in contact sports (Edelstein & Van Horn, 2023; Malcolm, 2023). The condition manifests as a gradual decline in cognitive function and can be accompanied by early indications such as memory loss, cognitive impairment, depression, and impulse control issues. In severe instances, the disease's gradual progression can result in dementia-like symptoms (Chen et al., 2023; Fu et al., 2023; Ryali et al., 2024) and, in extreme instances, self-harm (Bieniek et al., 2020; Mez et al., 2017).

Due to sports-related concussions being a complex and multifaceted condition, often accompanied by physiological, sociocognitive, and psychological symptoms (Furtner et al., 2017; Mateos-Pérez et al., 2018), it is important to note that clinical recovery, as measured by symptom resolution, does not necessarily equate to complete neurobiological recovery (D'Lauro et al., 2022). As mentioned, one of the key benefits of neuroinformatics-based approaches, such as ML, is the ability to identify non-linear relationships and high-order interactions between multiple variables, a result traditional statistics cannot produce (Bazarian et al., 2021). Considering a major limitation of identifying correlations between neurological disorders and concussions can be due to the large variance in the time elapsed since the last concussion and time of imaging, these models could obtain the ability to learn and interpret results despite the significant time lag, (Bergeron et al., 2019; Rosenblatt et al., 2021). With its advanced capabilities, ML has the potential to be the ideal solution for effectively addressing the intricacies of the neuropathological process of concussions in females (Fakhran et al., 2014; Jacob et al., 2022; Tamez-Peña et al., 2022). Thus, employing a multifactorial ML approach that considers the temporal patterns of various fluctuating biomarkers and alterations in cognitive and other functional metrics may be a superior method for identifying persistent concussion-related impairments or detecting the early stages of a chronic condition stemming from repeated head impact occurrences (Chen, 2018; Smith et al., 2019).

Current research in ML and sports-related concussion has primarily focused on male, adolescent, or adult athletes from team sports (Cowley et al., 2021; D'Lauro et al., 2022). While some studies have included mixed-gender samples, most participants reported being male. Thus, the lack of research on female athletes in ML for sports-related concussion management highlights the gender imbalance in sports and exercise science research (Dams-O'Connor et al., 2023). Although women are included in sports science research and concussion injury research, the number of female participants is significantly lower than that of male participants. This disparity seems more pronounced in ML studies for concussion management than in the broader field of sports and exercise. Conducting additional studies utilizing diverse athlete data for analysis through ML algorithms may help bridge this critical knowledge gap and improve the prevention and treatment of sports-concussion, ultimately benefiting athlete well-being.

The examination of neuroimaging for concussed female athletes has revealed a significant lack of research regarding neuropathology and ML, resulting in a critical gap in statistical information and pathology (McKee et al., 2023). This gap in research is particularly concerning given the increasing frequency of concussions in female athletes and the potential long-term health consequences that may arise from inadequate diagnosis and treatment. Addressing this issue requires immediate attention and resources to advance the field of neuroimaging and improve our understanding of the pathology of concussions in female athletes. The utility of neuroinformatics approaches here is important since risk of CTE in sports appears to be closely linked to an athlete's career duration and the frequency of brain injuries sustained (Stone et al., 2017). To achieve this, it is essential to prioritize research utilizing neuroinformatics approaches to develop a comprehensive framework for research, defining what sports-concussion ‘looks like in male as well as in female athletes, aimed at properly diagnosing and treating concussions in females.

## Conclusion

Sports-related concussions constitute a multifaceted condition, characterized by a diverse array of physiological, cognitive, and psychological symptoms. Importantly, it's essential to recognize that the mere resolution of clinical symptoms does not necessarily imply a complete neurobiological recovery. In this complex landscape, the power of ML emerges as a beacon of hope, primarily due to its unique ability to unearth non-linear relationships and high-order interactions among various variables, a feat that traditional statistical methods cannot achieve. This capability opens the door to a deeper understanding of the intricate nuances within sports-related concussion, potentially revolutionizing the way we approach its management, diagnosis, and treatment.

However, a critical issue looms large in the domain of sports-related concussion research: a glaring gender imbalance Nowatzki and Grant (2011). This gender disparity raises an urgent call to action to rectify this gap in sports-concussion research and clinical practice. To do so, the field of concussion research must embark on comprehensive research initiatives, rooted in foundational, evidence-based science, which encompass a diverse range of athlete data and employ neuroinformatics techniques to fully appreciate the intricacies of concussion and recovery in both male and female athletes. In essence, neuroinformatics is a critical tool in advancing our understanding of clinical syndromes stemming from sports-related concussions and can ultimately aid in the development of more effective prevention and intervention strategies.

This *call to arms* is not just a matter of academic concern; it holds profound implications for all athletes' well-being and long-term health outcomes. Concussion management, diagnosis, and treatment should be equitable and effective for all athletes, regardless of their sex or gender. Hence, a collective and concerted effort is imperative to harness the potential of neuroinformatics and advanced research methodologies to chart a path toward comprehensive, yet interpretable, sex- and gender-inclusive sports concussion studies.
